# Multivalent Aptamers: Versatile Tools for Diagnostic and Therapeutic Applications

**DOI:** 10.3390/molecules21121613

**Published:** 2016-11-25

**Authors:** Mariya Vorobyeva, Pavel Vorobjev, Alya Venyaminova

**Affiliations:** Institute of Chemical Biology and Fundamental Medicine, Siberian Branch of the Russian Academy of Sciences, Lavrentiev Ave., 8, 630090 Novosibirsk, Russia; vorobyev@niboch.nsc.ru (P.V.); ven@niboch.nsc.ru (A.V.)

**Keywords:** SELEX, aptamers, multivalency, design, conjugates, application

## Abstract

Nucleic acid aptamers generated through an in vitro selection are currently extensively applied as very valuable biomolecular tools thanks to their prominent advantages. Diversity of spatial structures, ease of production through chemical synthesis and a large variety of chemical modifications make aptamers convenient building blocks for the generation of multifunctional constructs. An opportunity to combine different aptamer functionalities with other molecules of interest such as reporter groups, nanoparticles, chemotherapeutic agents, siRNA or antisense oligonucleotides provides a widest range of applications of multivalent aptamers. The present review summarizes approaches to the design of multivalent aptamers, various examples of multifunctional constructs and the prospects of employing them as components of biosensors, probes for affinity capture, tools for cell research and potential therapeutic candidates.

## 1. Introduction

Aptamers are short single-stranded RNA or DNA molecules capable of tight and specific binding to their targets due to the formation of characteristic spatial structures. SELEX (Systematic Evolution of Ligands by EXponential enrichment) technology of aptamers’ isolation was proposed in the early 1990s by three independent research groups [1,2,3]. The outstanding progress achieved in this field in the next 25 years resulted in a large variety of selection methods (see [4] for a comprehensive review) and a huge set of aptamers specific to most diverse targets. Strong binding affinity and high specificity of aptamers make them an attractive alternative to monoclonal antibodies. Aptamers also offer a number of advantages over antibodies, such as an opportunity to generate an aptamer against almost any desired target (even toxic or non-immunogenic), longer shelf-life, stability in a wide range of conditions, and low toxicity and immunogenicity. The most prominent advantages of aptamers are: easy and cost-effective chemical synthesis and tolerance to different chemical modifications, including backbone modifications and conjugation with other molecules of interest. Thanks to these properties, aptamers can be rather easily modified in a site-specific manner to acquire high stability to nucleases or to modulate target binding affinity, and can be conjugated as well to reporter groups, cell-toxic molecules, nanoparticles, etc. (see recent reviews [5,6]). Until now, aptamers have attracted great attention as target-recognizing modules in the design of different bioanalytical systems [7,8,9] and for developing of specific therapeutic agents targeted to disease-related proteins, such as growth factors, chemokines and blood coagulation factors [10,11,12,13]. Although in therapeutic applications aptamers are still not as popular as monoclonal antibodies, their success story includes one FDA-approved drug Macugen, a number of aptamer drugs undergoing Phase II and III clinical trials and a lot of aptamers which are now at the pre-clinical stage (see [10,14,15,16] for review). Taking this into account, one can confidently assume that therapeutic aptamers will find much wider application in the near future.

Among the large variety of aptamer-based constructs, multivalent aptamers are of particular interest. The concept of multivalent aptamers was first proposed by Shi et al. [17] and proved by the example of the pentavalent RNA aptamer against splicing regulatory protein B52. Starting from the definition given in the first patent on multivalent aptamers [18], a multivalent aptamer can be defined as a construct composed of two or more identical or different aptamer motifs, with or without additional structural elements or functional groups. The very nature of nucleic acid aptamers suggests the idea to use them as LEGO^®^-like building blocks which can be joined together in a number of ways to generate structures having desired functions. As we mentioned above, a number of chemical modifications and conjugation methods are available for aptamers, thus supplying one with a rich toolset to design a tunable aptamer-based construct for a particular task. Simple concatenation of the same aptamer motif can significantly improve the avidity of a construct due to multiple target binding sites. Otherwise, the combination of different aptamer motifs offers great opportunities to build multifunctional molecules that can serve as a basis for multiple-analyte biosensors, therapeutics targeting the certain proteins or cells, cell-specific immunomodulators or theranostic agents. During the last decade, this area of aptamer science has gained increasing interest. This review focuses on the design of multivalent aptamers, the examples of bioanalytical and therapeutic constructs on their basis, and prospects of their application.

## 2. Design of Multivalent Aptamers: General Principles

A key point in the design of multivalent aptamers is the way of connecting individual aptamer modules to a construct. The concrete method of connection is determined by peculiarities of aptamer-target interaction and by the desired functions of a multi-aptamer construct. In principle, aptamer domains can be covalently fused in an end-to-end manner, i.e., with zero linkers, but this approach is quite rarely used [19,20,21,22]. As a rule, linkers of different length and nature are required for proper functioning of multivalent constructs.

In most cases, aptamer modules are joined by covalent linkages. The most obvious approach, guided by the nature of nucleic acid aptamers, is based on the use of oligonucleotide linkers. Varying the length and the sequence of such a linker, one can control the distance between individual aptamers and regulate their -interactions. If each aptamer unit is intended to function independently, linkers of ‘neutral’ type are used, such as oligo(A), oligo(U) or oligo(T) [20,23,24,25,26,27,28,29,30,31,32]. A somewhat more intricate way is to use the oligonucleotide linker derived from the sequence of a particular full-length aptamer that was shown to be non-essential for binding [28]. The length of “neutral” linkers varies from 3 [30] to 50 [23] nucleotides, and 15–20 nt length is the most typical.

A more reliable, but also more cumbersome approach to linker design requires an additional SELEX of the linker sequence [33,34]. This so-called “chimeric” SELEX includes the use of different combinations of aptamer domains together with the randomization of the linker. After several selection rounds, the population is enriched with molecules which retain binding activities of all monomers in the context of multivalent construct. This strategy was developed for the design of bivalent aptamers but, in principle, it could be employed to obtain more sophisticated multivalent molecules with a desired set of activities.

We would also like to mention here the effect of avidity for binding of multivalent aptamers with the targets that are themselves multivalent, (e.g., multimeric proteins or proteins expressed at high density on cell surface). The in-depth theoretical considerations of avidity effect for multivalent ligand-receptor interactions are given in [35]. While the term *affinity* characterizes an interaction between one aptamer domain with its binding site (assessed by corresponding dissociation constant K_D_), the *avidity* refers to the overall strength of multiple binding interactions and can be described by the K_D_ of the completely associated aptamer-target complex. When the target contains two or more aptamer-binding sites, the binding of one aptamer domain promotes the binding of yet more domains (positive cooperativity), and the overall avidity can significantly exceed the affinities of individual aptamers. However, to obtain the prominent avidity effect for multivalent aptamers, a smart design of the multivalent construct is required.

To achieve proper positioning of each aptamer domain within the multivalent construct, it was proposed to use elements of secondary structure, such as double-stranded fragments or three-way junctions. Xu and Shi [36] developed a general scheme for the design of multivalent RNA aptamers employing three types of structural elements for the connection: (1) three-way junctions to organize and present aptamers; (2) stems to adjust local stability and relative orientation of aptamers; and (3) stable small U-turns to maintain the continuity of the strand. The feasibility of this approach was also proved by engineering RNA-based synthetic transcription factor comprising RNA aptamer and non-aptameric functional RNA domains [37]. Moreover, in the case of the multivalent RNA aptamers targeting the heat shock factor HSF1 [38], the optimization of the lengths and the flexibility of the linkages between aptamer domains resulted in 100-fold enhancement of avidity. Notably, the use of such complex linkers fits as well for DNA-based constructs and allows proper functioning of individual aptamers even when fused into a rigid, circular DNA molecules [39,40].

For some tasks, it is necessary to turn on the activities of the aptamer modules not simultaneously, but sequentially. In this case, the design of structure switching aptasensors necessitates the incorporation of one aptamer sequence into another such that two aptamer domains are connected by a short unstable stem [41,42]. Otherwise, aptamers share the linker sequence, and binding of one aptamer domain to its target leads to structural rearrangement in such a way that the second aptamer adopts an active conformation [43,44].

It is also possible to use linkers of non-nucleotidic nature. Polyethylene glycol (PEG) linkers are made of hexaethyleneglycol residues (-(OCH_2_CH_2_)_6_-*p*-, typically referred to as Spacer 18) joined by phosphodiester bonds; linkers of this type can be considered as totally sequence-neutral analogs of oligonucleotide linkers. As usual, 8–10 Spacer 18 residues are optimal to provide high binding avidity to soluble [28,45,46] or even cell-surface [47] proteins. It is worth mentioning that, according to recent data, PEG itself can evoke allergic reactions (which are now presumed to be a reason of allergy responses during clinical trials of PEG-modified REG1 aptamer [12,48]), and so this modification is probably not the best choice for therapeutic aptamers.

Polyacrylamide backbone can be employed as well for an assemblage of multivalent aptamers [49,50,51]. For this purpose, acrydite groups are attached to the 5′-termini of oligonucleotides during solid-phase DNA synthesis. Traditional ammonium persulfate/TEMED polymerization [49] or photopolymerization [50,51] gives multi-aptamer nanostructures comprising 10 s of aptamer domains.

Multimers containing a large number of aptamer motifs are also obtained by the covalent attachment of individual aptamers to the surface of Au [52,53] or Ag [54] nanoparticles or to the outer surface of viral capsid [55,56]. This type of multimerization is characterized by a vast increase of binding avidity.

The use of non-covalent interactions for an assembly of multivalent aptamers is a bit less widespread; however, it yields encouraging results. Aptamers can be joined together by Watson-Crick base pairing of additional complementary linker sequences or bridging oligonucleotides [57,58,59,60,61]. More sophisticated approach was proposed by Tahiri-Alaoui et al. [62]: aptamer motifs are supplemented with additional sequences of naturally structured RNA elements—CopA and CopT—which can form a very stable complex. Biotin-streptavidin interactions are also employed for the design of multivalent aptamers [63,64].

Notably, the functionality of multivalent constructs can be further extended by additional non-aptameric functional modules, such as small-molecule chemotherapeutic agents [50,51] or photodynamic therapy [55], antisense oligonucleotides [50,51] or siRNA [57].

**Figure 1:**
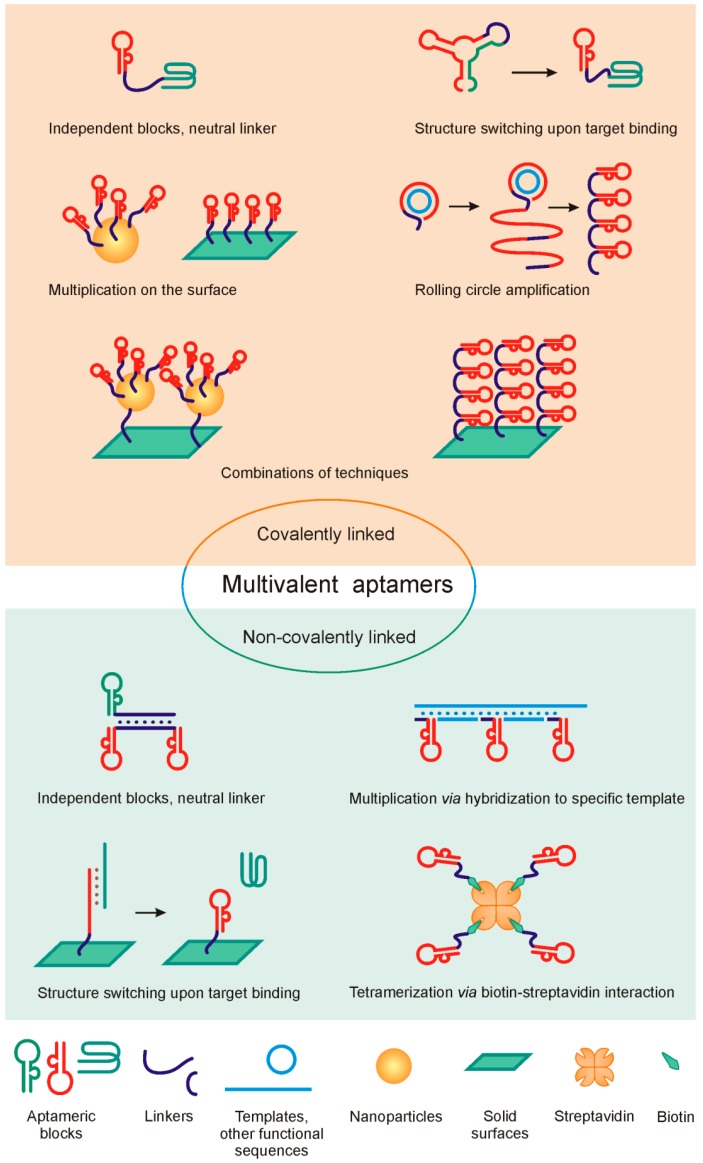
Schematic summary of approaches to multivalent aptamers design.

All the above-mentioned design strategies are schematically summarized in Figure 1. In the following sections we will consider their practical use in the context of bioanalytical and therapeutic applications of aptamers.

## 3. Analytical Applications of Multivalent Aptamers

### 3.1. Aptasensors Based on Multivalent Aptamers

Probably the most obvious variant of analytical application for bifunctional aptamers is a fusion of two aptamers: one capturing an analyte and the other binding to a reporting group. The key point of the design of these joint molecules is that the reporting group must be captured after the analyte is bound. Generally, such aptameric sensors (also reported as structure switching aptasensors) are designed using modular principle: connecting of two independent aptameric units by a short unstable stem. Binding of the analyte stabilizes the stem and the structure of the signaling aptamer. This makes possible the binding of the signaling dye or another molecule having a key role in the detection process.

Using this principle, a series of sensors to three different targets (ATP, theophylline, FMN) were designed in [41]. The sensors contained the aptamer to malachite green (MG) dye, that remarkably increases its fluorescence upon aptamer binding. All aptamers were shown to retain their original affinities to the targets. An interesting point of the study was the ATP sensor, which was made chimeric—i.e., consisted of DNA aptamer to ATP and RNA aptamer to MG. Two other sensors were completely RNA, which imposes the possibility of expression of such sensors within living cells.

Another example of modular design was published by Kato et al. [42]. A newly selected 76-mer DNA aptamer to dapoxyl fluorescent dye increased its fluorescence by more than 700-fold upon binding. This sequence was subjected to truncation and mutational analysis to find a proper site for the fusion with analyte-capturing aptamer. The obtained 42-mer aptamer motif was used to design two turn-on bivalent sensors, for thrombin and for ATP. Notably, for the ATP sensor the F/F_0_ parameter (fluorescence of the sensor in the absence/presence of the target) was significantly higher than for the analogous aforementioned sensor based on the MG-binding aptamer. The thrombin sensor with the best F/F_0_ value was shown to respond to 100–500 nM of thrombin in diluted fetal bovine serum, although the F_0_ parameter was slightly increased in these conditions, probably because of nonspecific binding of dapoxyl with serum proteins.

What is noteworthy is that the structure switching sensor design based on the secondary structure data generally leads to incorporation of one aptamer’s sequence into another. However, sequential conjugation of two aptamers is also possible. Chang et al. [26] reported structure switching bifunctional combined aptamer (BCA) made of two DNA aptamers, one to thrombin and another to streptavidin. The linking DNA sequence was designed in a way enabling for BCA to adopt inactive conformation towards both targets. Binding of thrombin triggered the conformational change which led to activation of streptavidin aptamer and subsequent immobilization on streptavidin-coated beads. The amount of bound BCA was proportional to the concentration of 5′-fluorescein label.

Analogously, a structure switching sensors for SPR analysis of interferon γ (INF-γ) were developed [65,66]. The amplified surface plasmon resonance aptasensor for INF-γ was based on a streptavidin-incorporated aptamer. The fused sequence of two aptamers formed stable hairpin structure which is inactive towards streptavidin. Some portion of the INF-γ recognizing sequence not involved in the hairpin was accessible for the target protein. Binding of INF-γ to its aptamer led to conformational rearrangement of the second aptamer, which in turn bound to streptavidin thus amplifying the biosensor’s response. The detection limit of these aptasensors was in the picomolar range.

An interesting group of aptasensors is represented by bifunctional aptamers to different analytes for their parallel detection. Analytical signal in this case can be generated in a number of ways. A dual sensor reported by Elbaz et al. [24], comprised two partially complementary DNA strands, one (sensing) being a joint of two aptamers against cocaine and AMP, another (signaling) a DNAzyme mimicking horseradish peroxidase. Binding of any of the analytes enabled the signaling strand to form active conformation, bind hemine and start peroxidase reaction. Other kinds of signal readout were Faradaic impedance spectroscopy and field-effect transistors (FET). In these cases, the blocking strand partially complementary to both sensing aptamer domains was covalently immobilized on the surface of the Au electrode or FET gate surface. Adding of any of the analytes led to the dissociation of sensing and blocking strands and a decrease of surface-associated negative charge which was registered as the analytical signal. The authors propose the use of bifunctional aptameric systems as elements of bioelectronic logic gate systems or signal transduction cascades.

Other examples of dual aptasensors include constructs recognizing a protein or a small molecule. Thrombin or ATP were detected by one sensor based on electrochemical impedance spectroscopy [67]. One unimolecular DNA strand comprising both aptamers was hybridized with a short DNA covalently linked to a gold electrode. Binding of any target molecule led to dehybridization of the sensing strand, which was registered electrochemically.

An electrochemiluminescent biosensor based on bifunctional aptamer to thrombin and adenosine showed excellent sensitivity to both targets and the ability to detect both targets in one plasma sample [68]. Multiple copies of the biotinylated bifunctional aptamer were immobilized on streptavidin-coated gold nanoparticles (AuNPs) attached to a gold electrode. *N*-(aminobutyl)-*N*-(ethylisoluminol) modified AuNPs were used for signal generation in connection with the small DNA-probe complementary to a portion of adenosine aptamer. Binding of adenosine released this probe and reduced the signal proportionally to the concentration of adenosine. For thrombin detection, bifunctional aptamer was also used as a capturing probe. The signal was generated by another thrombin-binding aptamer with the same electrochemiluminescent label. In this case, the signal was proportional to thrombin concentration.

Lysozyme or adenosine can be detected by cyclic voltammetry response of DNA-bound [Ru(NH_3_)_6_]^3+^ in an aptameric biosensor consisting of two corresponding DNA aptamers, hybridized but not linked covalently to each other [69]. Significant signal enhancement was achieved due to DNA-AuNP conjugates, connected with the sensor also by a complementary sequence.

Goda et al. [25] proposed to combine two different aptamers to one target for improved detection. In a potentiometric study of thrombin biosensing, two thrombin aptamers—TBA15 and TBA29—were tested separately or in combinations: both aptamers immobilized in a close proximity to each other or two aptamers joined by a linker and connected to an electrode. The authors concluded that sensors made of two different aptamers, either as a bifunctional construct or separately attached to the surface, were more efficient compared to mono-aptamer sensors. However, only a moderate difference can be seen from the K_D_ values (for example, at pH 6.0 the lowest K_D_ value for bivalent construct was 41 ± 32 nM whereas the highest K_D_ for monovalent aptamer was 89 ± 34 nM).

### 3.2. Multivalent Aptamers as Analytical Probes

Le et al. [21] designed a bifunctional aptamer incorporating streptavidin-binding domain for analytical purposes. A newly developed aptamer to streptavidin was conjugated sequentially with an aptamer to MG or theophylline, and the resultant constructs retained binding affinity both to streptavidin and small-molecule targets. A possibility of immobilization of bivalent aptamers on streptavidin-coated nanoparticles was also demonstrated. The authors suggest that this strategy of streptavidin immobilization of other aptamers represents an alternative to biotin labeling for a number of analytical techniques. Interestingly, a similar approach was employed earlier by Tahiri-Alaoui et al. [62]: 2′-F-RNA aptamer against streptavidin employed as a tag in bivalent construct (“adaptamer”) comprising also the aptamer specific to CD4+ cells. Two aptamers were joined non-covalently, via complementary CopA or CopT RNA elements. Such adaptamers were successfully employed to capture target cells onto a SA-derivatized surface.

Bivalent aptamers were used as an analytical probes in AFM experiments [69]. Using single-molecule force spectroscopy of immobilized bivalent aptamer consisting of thrombin aptamers to exosites 1 and 2, individual thrombin-unbinding forces for each part were determined. The linker influenced the interaction of aptamer to exosite 1 but not to exosite 2 with thrombin.

Bivalent constructs ‘chelating’ a target are regarded as ultra-high-affinity reagents, which can be employed in variety of analytical or therapeutic applications. Bivalent thrombin aptamers are the most popular example of the type (see also Section 4.1), although their monomer components were isolated independently by different research groups. Recently, an assay for generating pairs of aptamers which can be used in these bidentate constructs was presented by Cho et al. [27]. After microfluidic selection to human angiopoetin-2 and high-throughput sequencing, an aptameric DNA chip was prepared with a large set of selection leaders. Next, a high throughput screening was performed to reveal the pairs of aptamers binding to various epitopes. Subsequent covalent binding of two aptamers with a flexible (dT)_25_ linker generated a highly avid reagent (K_D_ = 97 pM), with approximately 200-fold enhancement in affinity relative to free components.

### 3.3. Multivalent Aptamers for Cell Studies

Another promising approach to the design of functional aptameric constructs is a multiplication of one aptamer to achieve the desired effect. Functional studies of cell pathways and mechanisms can require an expressible form of a multivalent aptamer. For example, Shi et al. [17] developed the expression system for the multivalent inhibitory RNA aptamer against B52 protein, a splicing regulator in *Drosophila melanogaster*. This aptamer was expressed both in cultured cells and in vivo and suppressed phenotypes caused by B52 overexpression.

Shui et al. [70] constructed expressible di-and tetrameric forms of RNA aptamer against GFP (which was also found to be specific to other fluorescent proteins). The authors suggest that such aptamers, which bind fluorescent proteins and affect their properties, markedly expand the possibilities of their use in the study of biological pathways.

**Figure 2:**
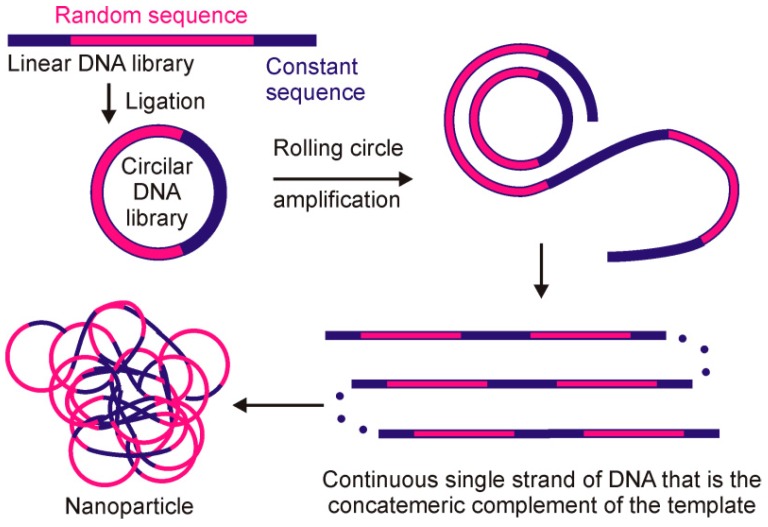
A general scheme of RCA-based DeNAno method for obtaining cell-specific DNA superstructures [71].

Selectable libraries of DNA nanoparticles can be generated by rolling circle amplification (RCA) of cyclized template containing random region. To this purpose, Steiner et al. [71] developed a new method called DeNAno to obtain DNA superstructures specific to human dendritic cells. Rolling circle replication of a circular DNA template using a strand displacing DNA polymerase produces a continuous single strand of DNA that is the concatemeric complement of the template. This ssDNA condenses into a discrete particle that can be visualized by fluorescent microscopy and flow cytometry if fluorescently labeled (see Figure 2 for the general scheme of the method). These cell targeting DNA nanoparticles, which in fact are multivalent aptamers, may found applications in cell imaging, cell sorting, and cancer therapy.

Recently, Kim et al. [72] proposed the multiplication of aptamers in complex non-covalent structures. Multiple copies of aptamer to mucin 1 (MUC1) were hybridized to a long polymeric RNA carrier obtained by chemical ligation of monomeric RNA units bearing thiol groups on both termini. Each RNA unit included two alternated sequences: one for aptamer binding, another for short probes, containing fluorescent dye and quencher. After an assembly of the whole system, the resultant multimeric MUC1 aptamer provided efficient internalization of the whole construct into cells; after that, short probes were displaced by cellular miRNA-34, giving the fluorescent signal proportional to the concentration of miRNA.

A multiplication of aptamers can be achieved as well via covalent immobilization on a surface of nanoparticle or microfluidic channel. Multiplication of fluorescently labeled aptamer to PTK 7 on gold nanorods resulted in significant fluorescence intensity enhancement at molecular recognition of CCRF-CEM cells [73]. The target cells can be clearly visualized due to multiplication of aptamer. This platform combines high signal level, high binding avidity (K_D_ = 0.085 nM vs. 2.24 nM for monovalent aptamer), and low non-specific binding, so it can be considered promising not only for cancer cell studies, but also for targeted therapy (due to strong infrared absorption of nanorods).

Oscoy et al. [74] generated very sophisticated gold-manganese oxide (Au@MnO) based nanostructures bearing multiple copies of cell-binding aptamer to PTK 7 and aptamers to ATP (as an example of intracellular metabolite). These constructs were shown to serve simultaneously as probes for intracellular capture of ATP and a matrix for consecutive matrix assisted laser desorption ionization mass-spectrometry [74].

Spherical gold nanoparticles were also used as a platform for aptamer multiplication [75]. The surface of a microfluidic device was modified with aptamer-coated AuNPs. Each nanoparticle was loaded by up to 95 aptamers on flexible PEG-based linkers. The efficiency of cell capture in this device was much higher than in a control device with uniformly linked monomeric aptamers. Depending on the type of aptamer multiplied on nanoparticles these microfluidic devices efficiently captured CCRF-CEM or Ramos cells from blood samples.

Zhang et al. [76] modified an inner surface of microfluidic channel with a mixture of aptamers and antibodies to protein tyrosine kinase 7 (PTK7), cell surface cancer biomarker. Capture of target CCRF-CEM cells was significantly improved as compared to assays based on antibodies alone or aptamers alone.

In addition to surface multiplication, rolling circle amplification of an aptamer can be easily performed using a circular synthetic template. ‘Unlimited’ extension of immobilized primers generates a kind of a network containing repeated binding units. Being inspired by mucus produced by many creatures in nature, which effectively captures food from the environment, Zhao et al. [77] proposed to use RCA multiplied aptamers to capture target cells in a microfluidic device. The aptamer to PTK7 was multiplied and then used for catching of lymphoblast CCRF-CEM cells. After effective concentrating of target cells, they can be released by enzymatic restriction of DNA network, and analyzed. This device is unique in efficiency of specific cell-capturing at high flow rates, easiness of captured cells release, and purity of target cells fraction.

A summary of analytical applications of multivalent aptamers is presented in Table 1. Taken together, the data summarized in the table demonstrate great opportunities opened by the use of multivalent aptamers for creation of very specific, sensitive, and tunable tools for quantitative detection of biomolecules, capture of proteins or whole cells, studies of biological pathways, and cell imaging.

## 4. Multivalent Aptamers as a Basis for Therapeutic Agents

### 4.1. Anti-Thrombotic Aptamers

Protein anticoagulants are widely used in clinical practice for a treatment of chronic and acute conditions associated with the risk of thromboembolism. The major drawbacks of such potent agents as heparin or bivalirudin are excessive bleeding, thrombocytopenia, and highly variable dose-response relationship. Aptamers represent a very promising alternative to protein anticoagulant as their effect can be quickly and reliably regulated by oligonucleotide antidotes [13].

The most popular protein target for anticoagulant aptamers is thrombin. A whole bunch of articles was devoted to the design of bi- or multivalent aptamers built of two anti-thrombin aptamers TBA15 and TBA29 targeting thrombin exosites I and II, respectively (briefly summarized in Table 2). Müller et al. [29] obtained a bivalent aptamer made of TBA15 and TBA29 connected by (dA)_15_ linker. Notably, this fused construct demonstrated only moderate improvement of binding affinity, but its thrombin-inhibiting activity in a blood clotting test was significantly enhanced as compared to precursor aptamer TBA15. A similar approach was applied by Hasegawa et al. [19,20]: anti-thrombin TBA15 and TBA29 were joined by more flexible poly(dT) linker. Bivalent aptamer containing (dT)_5_ linker demonstrated sub-nanomolar K_D_ value (1/10 of that for TBA29) based mainly on the much smaller dissociation rate. Thrombin inhibitory activity of bivalent aptamers also increased, at that longer linkers (up 20 dT residues) provided higher activity despite that K_D_ values for these linkers were somewhat higher; this observation is in agreement with the abovementioned results of Müller et al. [29]. Interestingly, the same principle of bivalent aptamer design was also applied in [20] to obtain constructs made of two identical anti-VEGF DNA aptamers (VEa5) targeting the heparin binding site of homodimeric VEGF protein. In this case, the most avid bivalent aptamer with zero linker demonstrated ~20-fold decrease of K_D_ as compared to monomer ancestor, while linkers of 5–20 dT residues provided very weak target binding. These results count in favor of individual design of multivalent aptamer system for every protein of interest.

It was also proposed to connect these two aptamers by flexible PEG linker. Kim et al. [45] examined the series of bivalent aptamers with different linker length (2–10 Spacer 18 residues). It was found that eight spacers (~16 nm) provide 62-fold K_A_ improvement due to lower dissociation rate, and nine-fold increase of clotting inhibition. Similarly, Tian et al. [46] connected TBA15 and TBA29 by 24 nm long PEG linker of 10 Spacer18 residues. This led to at least ~100-fold decrease of K_D_ as compared to monomer counterparts, and significantly enhanced anticlotting activities. The most efficient aptamer TBA15-(Spacer18)_10_-TBA29 was more active in clotting test than bivalirudin, a clinically used anticoagulant.

As an alternative to rational design, Ahmad et al. [34] proposed in vitro selection of the optimal nucleotide sequence joining TBA15 and TBA29. The length of the randomized linker region was 35 nt, since it spans the distance between two aptamer binding exosites, and contains enough nucleotides to fold into an optimal structure. After five selection rounds, the resultant 119-nt aptamer TBV-08 demonstrated ~200-fold improvement of binding affinity (K_D_ = 8.1 pM) as compared to monomeric aptamers, and a ~15-fold improvement as related to previously designed anti-thrombin bivalent aptamers. Interestingly, the secondary structures of all bivalent aptamers obtained by this method represented a dumbbell with two thrombin aptamers forming loops on each end, and a linker region interacting with primer-binding sites to form a rigid double-stranded stem. Within this structure, both aptamer modules are flexible enough to adopt an optimal conformation for target binding. TBV-08 was also found to be an efficient thrombin inhibitor in fibrinogen cleavage reaction.

A very elegant approach to anti-thrombin bivalent aptamer design was reported by Wang et al. [44]. As a rule, antidotes to aptamers are represented by complementary oligonucleotides, but the authors proposed to use hemin for the purpose. Bivalent DNA aptamer consisted of only 27 nucleotides and comprised two sequences: TBA15 and hemin-deoxyribozyme, sharing 6-nt sequence. In the presence of K^+^ ions and thrombin, TBA15 part formed an antiparallel G-quadruplex and binds to the cognate target, and the addition of hemin led to unfolding of bivalent aptamer, its releasing from thrombin, and re-folding to parallel G-quadruplex. A feasibility of this approach was proved by the three-fold increase of clotting time in human plasma in the presence of bivalent aptamer, and the restoration of initial clotting time upon the addition of hemin. Since heme compounds are now successfully employed as drugs, low cytotoxicity of this antidote is proven by years in clinical practice, which makes this anticoagulant-antidote pair even more promising for anti-thrombotic therapy.

Bivalent 2′-F-RNA aptamer anticoagulants targeting two proteins of coagulation cascade were recently reported by Soule et al. [30]. Inspired by the example of natural anticoagulant heparin which binds to multiple coagulation enzymes, bivalent constructs were made of prothrombin aptamer and the aptamer against Factor Xa connected by (pA)_3_ linker. The most efficient bivalent aptamer RNA_BA_4m retained high affinity to both targets and was able to anticoagulate human plasma, although clotting time was no higher than that for the mixture of individual aptamers. However, the authors emphasize that a single bivalent molecule is preferable from a safety and drug development point of view. Moreover, the effect of bivalent aptamer can be reversed by the single antidote oligonucleotide that binds to the junction site.

One of the first examples of therapeutic multivalent aptamers bearing more than two aptamer units was described by Di Guisto et al. [39,40]. To increase nuclease stability of aptamers and improve their binding properties, monomeric aptamer units were joined into circular multivalent constructs. Four different DNA aptamer motifs were employed as building blocks: anti-thrombin aptamers TBA15 and TBA29, L-selectin aptamer and the aptamer against red blood cell marker. A DNA hairpin acted as an ancillary module between aptamer units; all aptamer sequences contained additional flanking regions to form extended stem-loop structures. Each construct, assembled by means of DNA ligation, contained two, three or four identical or different aptamer motifs. A single three-way junction motif produced a three-headed aptamer, and two three-way junctions gave a four-headed construct. These circular aptamers possessed high serum and plasma stability (half-lives of several hours vs. <1 h for monomer aptamers). Their functional activities were also improved: anti-thrombotic multivalent aptamers made of TBA15 demonstrated two to three-fold higher potency in a clotting test as compared to monomer counterpart. Anti-thrombotic activity of circular aptamers was suppressed by antidote antisense oligonucleotides. Notably, antidotes could also be designed as circular constructs, although higher concentration of circular anti-aptamer was required for antidoting, most likely due to topological problems. The potential of innate immunity modulation was studied for circular multivalent aptamers, particularly their ability to stimulate an inflammatory response through toll-like receptor activation. Despite the presence of CpG dinucleotide motifs, the constructs cause only mild anti-inflammatory response, so the authors suggest that circular aptamers would not have major side effects upon innate immunity [40].

To optimize the inhibition of thrombin enzymatic activity, Hsu et al. [52] employed a physical multimerization of aptamers on the surface of Au nanoparticles. The conjugates that comprise TBA15 and TBA29 attached to the NP surface through Au-thiol interactions, and sulfated galactose acid (sulf-Gal). The latter component was intended to further improve the anti-thrombotic properties of the whole conjugate due to its own ability to inactivate thrombin. The resulting multivalent nanoparticles exhibited extremely high binding avidity (K_D_ = 3.4 fM)—over 100 times higher than that of monovalent TBA29; 10,000 times higher than that of TBA15, and at least 10 times higher than reported previously for fusion aptamers, dendritic aptamers, and TBA29–AuNPs; yet another example of the abovementioned avidity effect. The conjugate containing 15 TBA15 and 15 TBA29 molecules per one AuNP was the most effective in standard clotting tests, and retained its activity after 48 h of incubation in human plasma. AuNPs modified with oligonucleotide antidotes reversed the activity of anti-thrombotic conjugates.

Recently, the same research group [53] proposed a different approach for the design of aptamers multimerized on AuNP: a self-assembled hybrid monolayer strategy. Briefly, each building block contained an aptamer sequence (TBA15 or TBA29), a sequence for hybridization, and (dA)_20_ fragment responsible for anchoring on Au surface. Mixing of these “monomers” with AuNPs resulted in stable multivalent constructs containing approx. 30 aptamer functionalities (TBA15 and TBA29 hybridized through abovementioned sequences) on each NP. This new multivalent construct possessed femtomolar affinity to thrombin, and its inhibitory potency was 4.5-fold higher as compared to abovementioned 15-TBA15/TBA29/sulf-Gal–AuNP. Moreover, the inhibition of thrombin by the complex could be reversed after green laser excitation (532 nm) that triggers release of hybrid aptamer units from NP surface.

### 4.2. Anti-Inflammatory Aptamers

l-Selectin, which is expressed on the surface of most leukocytes, plays an important role in leukocyte trafficking in inflammation and injury. Nowadays, it is considered as potential target for new therapies of inflammatory disorders. A presence of carbohydrate recognition domain made it possible to generate multivalent synthetic oligosaccharide l-selectin inhibitors; however, they have some disadvantages such as complex manufacturing procedures and short half-life in vivo [81]. Alternatively, aptamer-based multivalent inhibitors of l-selectin were proposed. To generate a multivalent aptamer, Chang et al. [26] employed RCA from circular DNA template. The resultant DNA molecule contained approx. 30 monomers separated by (dT)_20_ stretches, and bound specifically to l-selectin with affinity greatly higher than that of monomeric aptamer. The multivalent aptamer inhibited the interaction of l-selectin on the cell surface with endogenous ligands, blocked cell homing in secondary lymphoid tissues in mice with nanomolar concentrations, and showed no cell toxicity during in vitro test. All these results made the construct developed by Chang et al. very promising drug candidate for a modulation of l-selectin signaling.

Very recently, Riese et al. [32] reported the creation of chemically synthesized dimer and trimer based on l-selectin binding DNA aptamer sequences separated by (dA)_9_ spacers, and bearing small PEG 5′-tails (PEG 281) to enhance blood stability. Both aptamers possessed picomolar target binding affinities and demonstrated leukocyte blocking in vitro. Trivalent aptamer effectively inhibited leukocyte rolling in model mice; thus it also looks very prospective in view of anti-inflammatory drug design.

### 4.3. Anticancer Aptamers

#### 4.3.1. Aptamers Binding to Cell-Surface Targets

A possibility to unite cell-specific aptamers and various cell-toxic agents in one multivalent construct opens a way to new therapeutic molecules for targeted killing of cancer cells. A large variety of such constructs was developed by different research groups.

Tong et al. [56] targeted receptor tyrosine kinase PTK7, a cancer-specific cell surface marker. Using the concept of multivalency, a genome-free capsid of MS2 bacteriophage was decorated by DNA aptamers specific to PTK7. Up to 60 aptamers were covalently attached to the capsid surface via specially developed oxidative coupling strategy. Briefly, an aptamer derivative bearing the phenylene diamine group interacted with an unnatural amino acid, *p*-aminophenylalanine, introduced into MS2 coat protein. These aptamer-modified capsids were specifically internalized into Jurkat cells expressing PTK7, and then trafficked to lysosomes. This supramolecular construct was then employed as a drug delivery vehicle for porphyrin, a photodynamic therapy agent [55]. A double mutant of MS2 was used, bearing sulfhydryl groups on the interior surface (for covalent attachment of porphyrin) and aniline groups on the exterior surface (for covalent attachment of aptamer derivative described above). The resulting modified capsid carried ~20 aptamers and up to 180 porphyrin residues. Such double-modified constructs were able to specifically target Jurkat cells and kill them after illumination. This strategy allows changing both aptamer and cytotoxic agent, so the whole multivalent construct can be tuned to meet desired cell specificity and cytotoxicity, and could also be adapted for cell imaging [56].

Receptor tyrosine kinase PTK7 was also targeted by Zhang et al. [31]. So-called Poly-Aptamer-Drugs represented multivalent aptamers obtained by RCA method, comprising of multiple copies of sgc8 DNA aptamer (30–40 aptamer units), separated by polyA linkers with 3 GC repeats. To make these constructs not only cell-specific but also cell-toxic, oligonucleotides complementary to linker sequences were hybridized with multivalent aptamers, and the resultant complexes were loaded with doxorubicin, DNA-intercalating chemotherapeutic agent (approx. 10 molecules per multivalent aptamer). Multivalent aptamers showed 40-fold improvement of binding affinity (in terms of K_D_) as compared to monomer counterparts, more efficient cell internalization and selective cytotoxicity against PTK7-expressing T-leukaemia CCRF-CEM cell line. It should be underlined that the possibility to vary the length and sequence of linker regions allow regulating the payload of the construct. Otherwise, the aptamer module can also be changed for targeting of the Poly-Aptamer-Drug to another cell type, so the whole construct is highly tunable. A possible bottleneck of the system is in vivo drug release. Since doxorubicin release occurs upon DNA hydrolysis by nucleases, multivalent constructs have to be stable in blood serum until bound to target cells which can require an additional DNA modification.

Yang et al. [49] developed cell-toxic multivalent aptamers against cancer cells using polyacrylamide as a backbone. Monomeric units for this type of constructs were 5′-acrydite derivatives of aptamers. The whole multivalent conjugate assembled from 5′-acrydite aptamer specific to one particular type of tumor cells (CCRF-CEM, K562 or Ramos cells, see Table 2 for more details) and dye-bearing reporting element, 5′-acrydite-T_10_-dye-3′. After a one-step polymerization procedure, the obtained multivalent chain contained ~90 aptamers. All polymeric conjugates demonstrated improved binding affinity towards cognate target cells. By virtue of reporter element, it was found that multivalent polymers are subjected to specific cell internalization. Notably, the constructs demonstrated selective cytotoxicity towards target cells, including drug-resistant cell line. Since multivalent aptamers derived by Yang et al. are suitable both for cell imaging and cell killing, they represent a very promising example of theranostic agents. The concept of acrydite-based assembly was further developed in recent works of the same research group [50,51]. A photo-polymerization of building units bearing acrydite groups together with a complex system of oligonucleotide connectors resulted in 3D-nanostructures of controllable size and shape, loaded with the aptamer which targets CCRF-CEM cancer cells and doxorubicin. These nanoconstructs showed efficient and specific cytotoxicity. Moreover, analogous nanostructures driven by KK1B10 aptamer were supplemented with MDR1 antisense oligonucleotide targeting P-glycoprotein, which is responsible for multiple drug resistance of cancer cells. These multifunctional nanocomplexes showed the specific cytotoxic effect on drug-resistant K562 cells.

The development of tumor-targeted aptamer-driven theranostic agents was also recently reported by Li et al. [54]. The authors applied the concept of aptamer multimerization on the surface of 50 nm silver nanoparticles (AgNP), somewhat similar to that described by Hsu et al. [52] for AuNP derived with thrombin aptamers (see above). A covalent conjugation with sgc8 aptamer targeting PTK7 receptor tyrosine kinase or TDO5 aptamer against heavy µ chains on the surface of Ramos cells led to functionalized AgNPs having an ability to induce specific apoptosis of target cells. If fluorescent derivatives of these aptamers are used for AgNP functionalization, the resultant conjugates can also be applied for specific cell imaging due to metal-enhanced fluorescence effect, while retaining their cytotoxic properties. The possibility to use such nanoparticles for simultaneous cancer therapy and cell images makes them very promising theranostic tools.

The multimerization of cell-targeting DNA aptamer against mucin 1 (MUC1), a membrane protein overexpressed in certain types of tumor cells, was employed by Yoo et al. [57] for addressed delivery of siRNA. The authors developed a design of multivalent comb-type aptamer-siRNA conjugates built of multimerized antisense strands connected by chemical ligation hybridized with aptamer-incorporating sense strands. The resultant rod-shaped multimer could be specifically recognized by cells and internalized by a clathrin-based endocytosis without any transfection agent. The aptamer-driven multimers inhibited target gene expression, while the analogous constructs comprising only multimeric siRNA were inactive, as well as monomeric siRNA-aptamer conjugates.

Multifunctional aptamers recognizing T-cell receptors are of special interest since they represent very potent tools to induce protective antitumor immune response. To modulate a T-cell receptor function, a simultaneous binding with oligomerized receptors on the cell surface is needed, so multivalent aptamers are particularly suitable for the task. A series of works of Gilboa and co-workers (see [82] for a detailed review) deals with the generation of multivalent 2′-F-RNA aptamers which can bind to co-stimulatory T-cell receptors and modulate their functions. For example, the aptamer Del60 against murine CTLA-4 receptor was selected in [58] and used as a basis of tetravalent aptamer. To assembly the tetramer, a double-stranded oligonucleotide linker contained four single-stranded ends acted as a scaffold bringing together four Del60 molecules with single-stranded 5′-tails. The length of the linker was chosen to correspond the distance between two CTLA-4 molecules on the cell surface. It was found that tetramers are at least 10-fold more potent inhibitors of CTLA-4 in cell culture as compared to monomeric form. Moreover, tetravalent constructs also inhibited tumor growth in mice.

RNA aptamer M12-23 specific to 4-1BB receptor with a completely different mechanism of action—the receptor agonist—was isolated and used for the design of bivalent construct [59]. To make a dimer, 3′-end of M12-23 aptamer was extended with either of two complementary sequences which can hybridize to form a 21-nt double-stranded linker. The given linker length (10 bp/helical turn) provides the correct orientation of both aptamers necessary to interact with a pair of 4-1BB receptors. While monomeric form of M12-23 was unable to deliver a costimulatory signal to T-cells, the bivalent aptamer sufficiently stimulated T-cell proliferation in vitro, and, being injected intratumorally, promoted tumor rejection in mice. A similar strategy was successfully applied to obtain a bivalent aptamer activating OX40 receptor [60]. In this case, two monomeric aptamers hybridized to DNA scaffold where two annealing sites were separated by PEG spacer providing an optimal distance between aptamers. It is worth pointing out that DNA scaffold was chosen to avoid the formation of dsRNA stretch that could potentially cause toll-like receptor activation.

To replace the intratumoral injection by addressed systemic administration, the bivalent construct can be supplemented with an additional aptamer module specific to cancer cells. A feasibility of this strategy was proved using the aptamer against human prostate specific membrane antigene (PSMA) as a targeting module [61]. The bi-specific construct comprised a dimer of two 4-1BB aptamers and anti-PSMA aptamer hybridized through complementary linker sequences. Systemic administration of the construct resulted in significant inhibition of PSMA-positive tumor growth in mice. However, the major current limitation of this strategy arises from the fact that co-stimulation occurs on the cell surface; so, the cancer-specific aptamer has to be targeted to cell-surface protein which is not internalized upon ligand binding.

In line with the abovementioned works of Gilboa’s group, Parekh et al. [63] developed the tetravalent DNA aptamer against CD30 receptor, a specific biomarker of certain lymphomas. Four DNA-biotin conjugates were brought together by means of streptavidin. The resultant tetramer specifically induced apoptosis in anaplastic large cell lymphoma cells, thus proving the potential of multivalent aptamers for cancer immunotherapy.

The strategy of bivalent aptamer-mediated tumor cell lysis implying the recruitment of natural killer (NK) cells was also developed by Boltz et al. [28]. Bivalent aptamers comprised two modules: (1) aptamers specific to CD16α, surface receptor expressed by NK cells; and (2) aptamers to c-Met receptor tyrosine kinase, a tumor-associated antigen. The constructs were synthesized as contiguous oligonucleotide chains including two minimized aptamer sequences joined by different linkers: (dA)_15_, short PEG chains or so-called ‘original’ linkers derived the fragments from parent full-length aptamer sequences shown to be non-essential for binding. The most effective aptamer bsA17, with the 7-heteronucleotide sequence as a linker, recognized both protein targets simultaneously, and demonstrated the greatest specific cytotoxicity against human gastric and lung cancer cells. Notably, all bivalent DNA aptamers showed rather high serum stability (half-lives > 6 h). The authors explained this by a rigid structure of aptamers which makes them more stable to nucleases as compared to less structured DNA with exposed termini.

#### 4.3.2. Aptamers Binding to Soluble Proteins

Multifunctional aptamers could be employed as well to target cancer-related soluble proteins. Combining different modules in one aptamer-based molecule, Dupont et al. [22] developed a very potent inhibitor of serine protease urokinase-type plasminogen activator (uPA), the mediator of cancer metastasis participating in cell migration and cell invasion processes. To inhibit all activities of this multifunctional protein, two 2′-F-RNA aptamers against different domains of uPA were joined into contiguous RNA transcript with an additional 8 nt 3′-terminal sequence. The peptide aptamer upain-1 specific to another uPA epitope (the catalytic site of serine protease domain) was then attached to the 3′-end of RNA transcript through periodate oxidation of 3′-ribonucleotide followed by the reaction with aminooxy peptide derivative. Interestingly, the bivalent construct containing two RNA aptamers inhibited the interaction of uPA with its cognate receptor, uPAR, and delayed (but not completely turned off) the plasmin-activated proteolytic processing of pro-uPA to uPA. After the conjugation with the upain-1 peptide, the obtained trivalent construct almost completely inhibited all uPA catalyzed reactions. Notably, the specific inhibitory activity of the peptide as part of trivalent molecule enhanced from micromolar to subnanomolar range. The authors suggest the proposed strategy of rational design of heterovalent inhibitors could be a potent alternative to siRNA technology for complete functional knockout of proteins.

Another example of anti-cancer multivalent aptamers inhibiting soluble regulatory proteins was reported by Zhao et al. [38]. A systematic rational design of bivalent RNA aptamers inhibiting the interaction of heat shock factor HSF1 with its cognate DNA promoter elements was performed. HSF1 plays an important role in supporting highly malignant cancers. In bivalent constructs RNA aptamer motif was repeated twice, separated by heteronucleotide linkers of different length and composition to vary the distance between the subunits and the flexibility of the linkage. The optimal partial single strand linker for this system contained 9 base pairs + 3 nt and decreased K_D_ value by two orders of magnitude as compared to monomer precursor. This observation points out again the importance of proper design for multivalent constructs.

Mallik et al. [81] developed a very interesting strategy of conscripting the complement system to neutralize certain secreted proteins by means of multivalent aptamers. An aptamer against complement protein joined with a target-specific aptamer would give a kind of adaptor that allows “tagging” of the target protein for subsequent phagocytosis. The proof-of-principle for this concept was demonstrated by an example of bivalent RNA construct composed of the aptamer against the opsonin C3b/iC3b and an aptamer specific to GFP which was used as a model target. The use of such an aptamer promoted specific and efficient transportation of GFP into the lysosomes of phagocytic cells. This strategy could be in principle further extended to the specific opsonization of cancer cells by targeting certain biomarkers on their surface.

### 4.4. Antiviral Aptamers

In the context of antiviral therapy, multivalent aptamers comprising several modules against key proteins of viral lifecycle can be considered as potent and specific antiviral agents. To inhibit the intracellular proliferation of hepatitis C virus, Umehara et al. [23] performed a rational design of bivalent aptamers against multifunctional viral NS3 protein. Two RNA aptamer inhibitors of helicase and protease activities of the protein were connected by long polyU stretches (40–50 nt). Most effective bifunctional aptamers NEO-35-s41 and G925-s50 inhibited the protease activity of the NS3 viral protein in HeLa cell culture; their ability for in vitro inhibition of the viral genome replication system due to anti-helicase activity was also demonstrated.

Very recently, Maier et al. [82] developed a new approach to inhibit the infection caused by New World hemorrhagic fever mammarenaviruses (NWM). Since the viral entry to human cells requires binding of viral Glycoprotein 1 (GP1) with transferrin receptor (hTfR), the authors proposed to block GP1-hTfR interactions by means of 2′-F-RNA aptamers specific to hTfR. A minimal 48 nt aptamer motif, Waz, was capable of inhibiting infection of human cells by hTfR-targeting NWM (EC_50_ ~ 400 nM). Notably, a trimerisation of the aptamer through biotin-streptavidin interactions led to the significant improvement of the inhibition (EC_50_ ~ 30 nM).

## 5. Conclusions

Engineering of specific multifunctional biomolecules for analytical and therapeutic purposes is now becoming increasingly popular. A number of such constructs were developed on the basis of monoclonal antibodies; among them we should mention bi-specific therapeutic antibodies (so-called diabodies) for cancer immunotherapy (see, for example, the review [83]). However, nucleic acid aptamers provide a much more convenient basis for design of multifunctional constructs due to their higher stability, greater tolerance to chemical modifications, and a possibility of chemical synthesis. A broad spectrum of chemical approaches is available to conjugate aptamer modules with each other and with other molecules of interest, so almost any desired requirements of a particular research task should be met. A large variety of bioanalytical tools for biosensing, affinity capturing, and cell imaging (see Table 1) were developed by combining different aptamer modules and reporter groups, nanoparticles, or analytical devices. Multivalent aptamers and constructs on their basis also recommended themselves as very promising agents which could be used in prospective as anticoagulants, anti-inflammatory, and anti-cancer therapeutics (see Table 2).

To summarize, multivalent aptamers represent very potent and versatile platform which can be used for different bioanalytical and therapeutic applications. The area of such applications and the set of newly designed multivalent aptamer constructs will undoubtedly be expanded in the foreseeable future.

## Figures and Tables

**Table 1:** Summary of multivalent aptamer constructs for bioanalytical applications.

Target	Backbone	Aptamer Domains	Connection	Advantage of Multivalency	Reference
**Biosensors**					
ATP, theophylline, FMN	DNA, RNA	MG + ATP, MG+ theophylline, MG + FMN	Covalent; secondary structure, 2–4 bp stem	Fluorescent detection of the analyte	[41]
Thrombin, ATP	DNA	Thrombin+ dapoxyl, ATP + dapoxyl	Covalent; secondary structure, 1–4 bp stem	Fluorescent detection of the analyte	[42]
Thrombin	DNA	TBA15, streptavidin	Covalent; 4 nt pyrimidine sequence	Immobilization via streptavidin upon binding of thrombin	[43]
INF-γ	DNA	INF-γ, streptavidin	Covalent; no linker or (dT)_5_ or (dT)_10_	Amplified SPR detection of INF-γ	[65]
Cocaine, AMP	DNA	Cocaine, AMP	Covalent; (dT)_9_	Simultaneous detection of two analytes, functional assembly for logic gate “OR” operation	[24]
Thrombin, ATP	DNA	TBA15, ATP	Covalent; no linker	Label-free EIS detection of two analytes	[67]
Thrombin, adenosine	DNA	TBA15, adenosine	Covalent; no linker	Detection of two analytes	[68]
Lysozyme, adenosine	DNA	Lysozyme, adenosine	Non-covalent; assembled by hybridization of linker sequences	Detection of two analytes	[69]
Thrombin	DNA	TBA15, TBA29	Covalent; no linker or (dT)_5_ or (dT)_10_	Enhancement of the overall binding ability	[25]
**Analytical Probes**					
Streptavidin, MG, theophylline	RNA	Streptavidin + MG, Streptavidin + theophylline	Covalent, no linker	Streptavidin immobilization of aptamers	[21]
Thrombin	DNA	TBA15, TBA27	Covalent; unspecified 8-unit spacer	AFM study of unbinding dynamics and dissociation energy landscape	[78]
Human angiopoetin-2	DNA	Two aptamers to distinct epitopes	Covalent; (dT)_25_	~200-fold affinity enhancement	[27]
**Imaging**					
GFP	RNA	GFP	Covalent; 5S rRNA three-way junction	Enhanced binding and fluorescence modulation	[70]
Human dendritic cells	DNA	Library of multiplied random blocks	RCA	Selectable library of multivalent nanoparticles	[71]
Mucine-1	DNA	MUC1	Non-covalent; multiplication via hybridization with multimeric template	Efficient internalization	[72]
CCRF-CCM cells	DNA	Sgc8c	Covalent; SH-mediated nanorod surface multiplication	Co-stimulation of T-cells in vitro Tumor rejection in vivo	[73]
K-562 cells	DNA	KK1HO8	Covalent; SH-mediated nanorod surface multiplication	Enhanced cell imaging and targeting	[73]
**Affinity Cell Capture**					
CD4, streptavidin	RNA	SA19, CD4 aptamer	Non-covalent; via CopA-CopT interactions	Affinity capture of CD4+ cells	[62]
CCRF-CCM cells, ATP	DNA	Sgc8c, ATP	Covalent; on Au@MgO nanoflowers	Intracellular capture of ATP for subsequent MALDI analysis	[74]
CCRF-CCM cells or Ramos cells	DNA	Sgc8c or TD05	Covalent; via spherical AuNPs multiplied on microfluidic channel	High efficiency, throughput, and purity of cell capture from blood samples	[75]
CCRF-CCM cells	DNA	Sgc8c	Covalent; multiplication on microfluidic channel	Enhanced capture efficiency	[76]
CCRF-CCM cells	DNA	Sgc8c	Covalent; RCA multiplied aptamers immobilized on microfluidic channels	Highly efficient specific isolation of target cells from blood samples	[77]

**Table 2:** Summary of multivalent aptamer constructs for therapeutic purposes.

Target	Backbone	Aptamer Domains	Connection	Advantage of Multivalency	Reference
**Anticoagulants**					
Thrombin	DNA	TBA15, TBA29	Covalent; (dA)_15_	~2-fold K_D_ decrease ^1^; prolonged clotting time	[29]
Thrombin	DNA	TBA15, TBA29	Covalent; (dT)_20_	10-fold K_D_ decrease ^2^; ~3-fold increase of clotting time	[19,20]
Thrombin	DNA	TBA15, TBA29	Covalent; PEG, (Spacer 18)_8_	~62-fold K_A_ increase ^3^; ~9-fold increase of clotting time ^3^	[45]
Thrombin	DNA	TBA15, TBA29	Covalent; PEG, (Spacer 18)_10_	~100-fold K_D_ decrease ^1^, ~2.5-fold increase of clotting time ^1^	[46]
Thrombin	DNA	TBA15, TBA29	Covalent; in vitro selected 35 nt sequence	~200-fold K_D_ decrease ^1^; markedly improved inhibition of fibrinogen cleavage	[34]
Thrombin, hemin	DNA	TBA15, hemin deozyribozyme	Covalent; shared 6-nt sequence	3-fold increase of clotting time, restored upon hemin addition	[44]
Prothrombin, factor IXa	2′-F-RNA	R9D-14t, 11F7t	Covalent; (rA)_3_	Clotting time nearly the same as for the mixture of aptamers. Bivalent molecule is preferable for drug development. Effect reversed by oligonucleotide antidote	[30]
Thrombin	DNA (circular form)	TBA15, TBA29	Covalent; DNA hairpin	High serum and plasma stability; 2–3 fold increase of clotting time Effect reversed by oligonucleotide antidote	[39,40]
Thrombin	DNA	TBA15, TBA29	Covalent; attachment to AuNP; 15 TBA15 and 15 TBA29 per NP	100–10,000-fold K_D_ decrease ^3^; 10-fold increase of clotting time. Superior to commercial anticoagulants. Effect reversed by oligonucleotide antidote	[52]
Thrombin	DNA	TBA15, TBA29	Non-covalent; Attachment to AuNP by means of anchoring (dA)_20_ tail; 30 TBA15 and 30TBA29 per NP	10–1000-fold K_D_ decrease ^3^; Superior to commercial anticoagulants in clotting test. Superior to heparin in rat bleeding test. Effect reversed by green light irradiation	[53]
**Anti-inflammatory**					
l-Selectin	DNA	LD201*	Covalent; (dA)_9_; Trimer	10-fold increase of IC_50_ for l-selectin-ligand interaction. Inhibition of target cells’ homing in vivo	[32]
l-Selectin	DNA	LD201	Covalent; (dT)_20_; ~30 aptamer units per molecule	10^3^-fold higher affinity to l-selectin. More strong binding with l-selectin on cell surface. Inhibition of target cells’ homing in vivo	[26]
**Anti-cancer**					
CCRF-CEM cells (PTK7)	DNA	Sgc8c	Covalent; attached to MS2 capsid; up to 60 aptamer units	Target cell internalization. Addressed delivery of porphryin for photodynamic therapy	[55,56]
CCRF-CEM cells (PTK7)	DNA	Sgc8c	Covalent; PolyA linker with 3 GC repeats; 30–40 aptamer units. Loaded by doxorubicin	40-fold K_D_ improvement; More efficient cell internalization. Cytotoxicity against CCRF-CEM cells	[31]
CCRF-CEM cells (PTK7) K562 cells Ramos cells (IgM heavy mu chain)	DNA	Sgc8c or T2-KK1B10, or TD05	Covalent; polyacrylamide backbone; ~90 aptamer units	Improved binding affinity towards target cells; simultaneous cell imaging and cell killing	[49]
CCRF-CEM cells (PTK7) K562 cells	DNA	Sgc8c or T2-KK1B10	Covalent (polyacrilamide backbone) + non covalent (oligonucleotide connectors; Loaded by doxorubicin and anti-MDR1 oligonucleotide	Selective cytotoxicity, including drug-resistant cell line	[50,51]
CCRF-CEM cells (PTK7) Ramos cells (IgM heavy mu chain)	DNA	Sgc8c or TD05	Covalent; Conjugated with AgNP. Loaded with fluorescent dye	Cytotoxicity, cell imaging	[54]
MCF-7 cells (MUC1)	DNA	MUC1 aptamer	Non-covalent; comb-like construct. Conjugates of aptamer and sense siRNA strand hybridized to multimerized antisense strand	Specific cell binding and internalization. Inhibition of target gene expression	[57]
CTLA-4 T-cell receptor	2′-F-RNA	Del60	Non-covalent; tetramer assembled on dsDNA linker	Enhanced bioactivity. Inhibition of tumor growth in vivo	[58]
4-1BB T-cell receptor	2′-F-RNA	12–23	Non-covalent; dimer assembled by hybridization of linker sequences	Co-stimulation of T-cells in vitro. Tumor rejection in vivo	[59]
OX40 T-cell receptor	2′-F-RNA	9.8	Non-covalent; dimer assembled on DNA scaffold	Co-stimulation of T-cells in vitro. Tumor rejection in vivo	[60]
4-1BB T-cell receptor PSMA	2′-F-RNA	12–23 xPSM-A10	Covalently linked 12–23 dimer; hybridized with xPSM-A10 through linker sequences	Inhibition of PSMA-positive tumor growth in vivo upon systemic delivery	[61]
CD30 T-cell receptor	DNA	C2	Non-covalent; biotin-streptavidin interactions; tetramer	Induction of receptor oligomerization and apoptosis of target cells	[63]
CD16α receptor of NK cells c-Met receptor of PBMC cells	DNA	CD16- α aptamer, C-met aptamer (different combinations)	Covalent; (dA)_15_, PEG or ”original” oligonucleotide linkers	Simultaneous binding of both target proteins. Target cell lysis	[28]
Urokinase-type plasminogen activator	2′-F-RNA/peptide	upanap-12, upanap126, upain-1	Covalent; zero linker between nucleic acid aptamers; 3′-conjugate with peptide aptamer	Complete inhibition of UPa processing and catalytic activities	[22]
Heat shock protein HSF1	RNA	AptHSF-RA1	Covalent; oligonucleotide linker	100-fold improvement of binding affinity	[38]
Opsonin C3b/iC3b, GFP	RNA	AptC3-1, AptGFP-AP3	Covalent; double-strand oligonucleotide linker	Specific opsonization of GFP (model protein) into phagocytic cells	[79]
**Antiviral**					
NS3 protein of hepatitis C virus	RNA	NEO-III-14U or G9-II-20U, #5	Covalent; U_40_–U_50_ linkers	Inhibition of both helicase and protease activities of NS3 protein	[23]
Human transferrin receptor	2′-F-RNA	Waz	Non-covalent; biotin-streptavidin interactions	~10-fold increase of EC_50_ for inhibition of NWM infection in human cells	[80]

^1^ as compared to TBA29 and TBA 15, respectively; ^2^ as compared to TBA29; ^3^ as compared to TBA15.
